# Prospective comparison of non-invasive risk markers of major cardiovascular events in COPD patients

**DOI:** 10.1186/s12931-017-0658-y

**Published:** 2017-09-29

**Authors:** Jorge Zagaceta, Gorka Bastarrika, Javier J. Zulueta, Inmaculada Colina, Ana B. Alcaide, Arantza Campo, Miguel Divo, Ciro Casanova, José M. Marin, Victor M. Pinto-Plata, Bartolome R. Celli, Juan P. de-Torres

**Affiliations:** 1 Pulmonary Department, Clinica Universidad de Navarra, Av Pío XII, 36, 31008 Pamplona, Spain; 2 Radiology Department, Clínica Universidad de Navarra, Pamplona, Spain; 3 Internal Medicine Department, Clinica Universidad de Navarra, Pamplona, Spain; 4 Pulmonary Division, Brigham and Women’s Hospital, Boston, USA; 5 Pulmonary Department, Hospital Universitario La Candelaria, Universidad de La Laguna, Santa Cruz de Tenerife, Spain; 6 Respiratory Service, Hospital Universitario Miguel Servet, Zaragoza, Spain; 7 Baystate Medical Center, Springfield, MA USA; 8 Universidad de Piura, Piura, Peru; 9 Clínica Angloamericana, Lima, Peru

**Keywords:** COPD, Cardiovascular Risk, Framingham score, SCORE

## Abstract

**Background:**

Chronic Obstructive Pulmonary Disease (COPD) is an independent risk factor for cardiovascular (CV) disease, one of the most frequent causes of death in COPD patients. The goal of the present study was to evaluate the prognostic value of non-invasive CV risk markers in COPD patients.

**Methods:**

CV risk was prospectively evaluated in 287 COPD patients using non-invasive markers including the Framingham score, the Systematic Coronary Risk Evaluation (SCORE) charts, coronary arterial calcium (CAC), epicardial adipose tissue (EAT), as well as clinical, biochemical and physiological variables. The predictive power of each parameter was explored using CV events as the main outcome.

**Results:**

During a median follow up of 65 months (ICR: 36–100), 44 CV events were recorded, 12 acute myocardial infarctions (27.3%), 10 ischemic heart disease/angina (22.7%), 12 peripheral artery disease events requiring surgery (27.3%) and 10 strokes (22.7%). A total of 35 CV deaths occurred during that period. Univariable analysis determined that age, hypertension, CRP, total Cholesterol, LDL-Cholesterol, Framingham score and CAC were independently associated with CV events. Multivariable analysis identified CAC as the best predictor of CV events (HR; 95%CI: 1.32; 1.19–1.46, *p* < 001).

**Conclusions:**

In COPD patients attending pulmonary clinics, CAC was the best independent non-invasive predictor of CV events. This tool may help evaluate the risk for a CV event in patients with COPD. Larger studies should reproduce and validate these findings.

## What is the key question?

COPD patients are at high risk of major cardiovascular (CV) events. Several non-invasive CV risk markers have been proposed to predict these events in the general population but never been compared in COPD patients.

## What is the bottom line?

Coronary Artery Calcium (CAC) is the best non-invasive CV risk marker that predicts major CV events in this population of COPD patients.

## Why read on?

The incorporation of this tool into daily practice of practitioners caring for patients with COPD could help detect high risk patients for future major CV events.

## Background

Chronic Obstructive Pulmonary Disease (COPD) and cardiovascular disease (CV) are two of the leading causes of death in the world [1]. Several studies have demonstrated the close relationship between COPD and CV disease [2, 3]. Patients suffering from coronary artery disease (CAD) that also have COPD, double the risk of death from CV disease compared to patients without COPD [3]. On the other hand, patients with COPD are at greater risk of morbidity and mortality from CV disease [4, 5]. According to several studies CV deaths are amongst the major causes of death in patients with COPD especially in those with mild to moderate degree of airway obstruction [6–9].

Although the recent update of the COPD GOLD guidelines recommend an evaluation for the presence of associated comorbidities [10], they do not specify how to evaluate pre-symptomatic patients through the use of non-invasive tools. The guidelines only state that clinicians should use the same methodology used in patients without the disease, but the potential use of non-invasive tools in the evaluation of future risk for CV events in these patients has not been investigated. A recent systematic review and meta-analysis [11] highlighted the need for the development of strategies to screen for CV risk factors in patients with COPD.

In order to help clinicians caring for COPD patients better detect risk of future CV events, we decided to evaluate the ability of different non-invasive tools in predicting cardiovascular events in a population of real-life patients with COPD attending pulmonary clinics.

## Methods

**Fig. 1:**
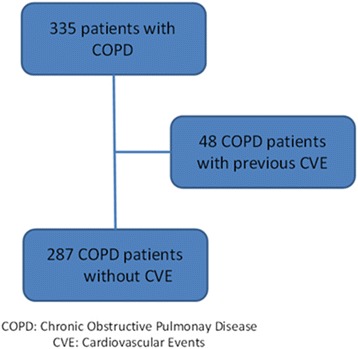
Flow diagram of the enrolment process and the patients included in the study

Participants were former and current smokers of at least 10 pack-years with previous spirometric diagnosis of COPD and regularly seen in our pulmonary clinic. All patients signed the consent form previously approved by the Human Review Board (Pamplona: “Comité de Etica de la Investigación, Universidad de Navarra IRB n°: 043/2006”). Subjects were consecutively enrolled from January 2006 to March 2016. COPD was confirmed by history and a post-bronchodilator Forced Expiratory Volume in the first second (FEV_1_)/Forced Vital Capacity (FVC) ratio of less than 0.7. All post bronchodilation measurements were performed 15 min after the inhalation of 400 μg of albuterol. To be enrolled, COPD patients had to be clinically stable and without exacerbations for 8 weeks prior to entry, and receiving optimal therapy according to international guidelines [10]. Exclusion criteria were uncontrolled co-morbidities such as malignancy or other confounding diseases. We recorded their previous history of diabetes mellitus (DM), hypertension (HTA), dyslipidemia, and the use of anti-hypertensive medications or statins. Blood pressure was measured following standard recommendations [12]. Patients with a history of a previous major CV event: acute myocardial infarction (AMI), ischemic heart disease/angina events or peripheral artery disease required surgery or a stroke, were not included in the study as shown in Fig. 1.

### Clinical variables

Lung volumes and spirometry were measured according to the ATS/ERS guidelines [13]. The 6-min walking distance (6MWD) was selected from the best of two walks separated by at least 30 min as recommended by the guidelines [14] (21). Modified Medical Research Council (MMRC) scale [15] evaluated patients` dyspnea. Body Mass Index (BMI) was calculated in kg/m [2]. FEV_1_% of predicted values, BMI, 6MWD and MMRC values were integrated into the BODE index [16] as previously described.

### Cardiovascular risk assessment scores

The validated tools explored in this study are summarized in the on-line supplement. They included the Framingham score and the Systematic Coronary Risk Evaluation (SCORE) charts [17, 18]. In brief, these scores include anthropological details, smoking, presence of DM, lipid profile and blood pressure.

### Laboratory measurements

Morning fasting blood and spot-urine samples were collected simultaneously while at rest and before any other test. The microalbuminuria (MAB) or urinary albumin excretion was determined as the urine albumin (milligrams) to creatinine (grams) ratio in the morning urine. Urine albumin concentration was determined by a standard turbidometric method (coefficient of variation 5.5%). Serum and urine creatinine concentrations were analyzed using the Jaffe reaction and quantified by a photometric method. Fasting serum levels of glucose, high sensitivity CRP, glycated haemoglobin (HbA1c) and cholesterol were also determined.

### CT Image acquisition and reconstruction protocol

All individuals were enrolled in a lung cancer screening program [19] and underwent a low radiation dose chest CT examination using a multidetector CT system (Somatom Definition and Somatom Sensation 64, Siemens Healthcare, Forchheim, Germany). Low-dose chest CT was performed with 120 kV, 40 mAs, 32 × 0.6 mm detector collimation, 64 × 0.6 mm slice acquisition, 0.5 s gantry rotation time, and 1.4 pitch. Images were reconstructed with 5-mm slice thickness using soft-tissue convolution kernel (B31f). No intravenous contrast was administered.

### Epicardial Adipose Tissue (EAT) Quantification

EAT volume was assessed by two independent observers (JZ, GB) unaware of the clinical information, using a commercially available software tool (Volume, Siemens) based on attenuation-dependent segmentation methods. Concordance coefficient between observers was 0.95; (95% CI: 0.93–0.96). Observers manually traced the pericardium at its superior extent (the center of the right pulmonary artery as it crosses the mid-sagittal plane), at mid-ventricular level, and at the end of the pericardial sac, which defined the inferior extent of the pericardial volume. The software automatically interpolated between the user-defined traces. Automatically traced contours were manually adjusted to the pericardium if needed. Epicardial adipose tissue volume was defined as any fat tissue located within the pericardial sac. A predefined threshold of −195 to −45 HU was used to identify voxels corresponding to fat [20].

### Coronary Calcium Calcification Evaluation

Each of the four main coronary arteries was identified (left main, left anterior descending, circumflex, and right). Calcification in each artery was categorized as absent, mild, moderate, or severe and scored by the radiologist as 0, 1, 2, or 3, respectively. Calcification was classified as mild when less than one-third of the length of the entire artery showed calcification, moderate when one-third to two-thirds of the artery showed calcification, and severe when more than two-thirds of the artery showed calcification. With four arteries thus scored, each subject received a coronary arterial calcium (CAC) score ranging from 0 to 12 [21].

### Outcome adjudication

Major CV events (AMI, ischemic heart disease/angina events, peripheral artery disease required surgery or a stroke) or deaths were registered reviewing each patient chart or contacting their family members.

### Statistical analysis

Data was summarized as relative frequencies for categorical variables, mean (SD) for normally distributed variables and median (25th–75th percentile) for non-normal data. To explore the association between Framingham score and CV event we used Spearman’s correlation coefficient.

To determine which factors best predicted the risk of developing a CV event, a Cox analysis was used. We first performed a univariable analysis with CAC, age, hypertension, total cholesterol, LDL-cholesterol, Framingham score, and SCORE charts and then a multivariable analysis including those that were statistically significant in the univariate analysis (*p* < 0.05). Because most of the factors found to be independently associated with CV events in the univariable analysis are included also in the Framingham score, we only compared this score and CRP levels with CAC in the multivariable analysis.

Significance level was established as a two-tailed *p*-Value ≤0.05. Calculations were made with SPSS 15.0, Chicago, U.S.A.

## Results

Figure 1 shows the flow diagram of the enrolment process. From the initial sample of 350 former and current smokers with COPD, we were able to enroll 287 patients without a previous history of CV events. All degrees of airway obstruction severity were included. The clinical, radiological and physiological characteristics of the patients are shown in Table 1.

**Table 1:** Clinical, radiological and physiological characteristics of the patients

Patients characteristics	
	COPD
N	287
Age (years)	64 ± 9
Follow up (months)	65 (36–100)
Gender (%) male/female	83/17
Pack-year (units)	51 ± 27
Current Smoker (%) yes/no	50/50
Framingham index (%)	27 (17–42)
SCORE index (%)	4 ± 3
Charlson (Units)	1 (1–2)
BMI (Kg/m2)	27.3 ± 4.8
FEV_1_/FVC (%)	55 ± 11
FEV_1_ (liters)	2.18 ± 0.8
FEV1%	70 ± 22
FVC %	99 ± 20
TLC %	106 ± 15
IC/TLC	0.33 ± 0.1
MMRC 0–4 (%)	36/32/21/9/2
6MWD (m)	465 ± 114
BODE Quart 1–4 (%)	82/10/5/3
Hypertension (%) yes/no	36/64
Anti-hypertensive treatment (%) yes/no	65/35
SBP (mmHg)	129 ± 19
DBP (mmHg)	76 ± 10.4
DM (%) yes/no	11/89
Glucose (mg/dL)	97 (90–108)
HbA1c %	5.9 (5.6–6.5)
Dyslipidemia (%) yes/no	71/29
Anti-hyperlipidemia treatment (%)yes/no	73/27
Total Cholesterol (mg/dL)	202 (172–232)
LDL-C (mg/dL)	124 (97.3–156)
HDL-C (mg/dL)	50 (41–61.8)
Urine Albumin/Creatinine ratio (mg/g)	12.8 (6–32.6)
EAT (cm^3^)	153 (112–211)
Coronary Calcium Score (Units)	2 (1–3)
CRP (mg/dL)	0.4 (0.2–0.8)
Systemic corticosteroid treatment (%) yes/no	6/94

*n* Number of participants, *BMI* Body Mass Index, *FEV*_1_ Forced Expiratory Volume in the first second, *FVC* Forced Vital Capacity, *TLC* Total Lung Capacity, *MMRC* Modified Medical research Council, *6MWD* 6 Minute Walk Distance, *BODE index* BMI, Obstruction, Dyspnea, Exercise, *SBP* Systolic Blood Pressure, *DBP* Dyastolic Blood Pressure, *DM* Diabetes Mellitus, *LDL-C* Low Density Protein, *HDL-C* High Density Protein, *EAT* Epicardial Adipose Tissue, *CRP* C reactive Protein

X ± SD means ± Standart Deviation, *p25-p75* interquartile range

The median follow-up time was 65 months (interquartile range: 36–100 months), and during this period 44 CV events were recorded: 12 (27.3%) acute myocardial infarction (AMI), 10 (22.7%) ischemic heart disease/angina events, 12 (27.3%) peripheral artery disease events requiring surgery and 10 (22.7%) strokes. In addition 35 deaths occurred during this period, 6 (17.5%) due to cardiac causes, 16 (46%) due to respiratory causes, 4 (11%) due to lung cancer, 5 (14.5%) due to other cancers, and 4 (11%) from other causes.

No differences were found in FEV_1_ in L (1.99 ± 0.7 vs 1.98 ± 0.7, *p* = 0.93) or % predicted (70 ± 22 vs 70 ± 21, *p* = 0.96) between those with and without a CV event respectively.

Table 2 shows the proportion of patients in each Framingham Score Risk category according to their spirometric GOLD groups. As expected, the risk of developing a CV event of a COPD patient is highly independent of their degree of airflow limitation. The small number of subjects included in grade 4, does not allow to obtain solid conclusion. The correlation between the Framingham score and the number of CV events was weak (*r* = 0.19) although statistically significant (*p* = 0.001).

**Table 2:** Proportion of patients in each Framingham Score Risk Category

GOLD spirometric category	Framingham Score Risk Category		
	<10%	10–20%	>20%
1 FEV_1_% > 80 (*n* = 109)	9	21	70
2 FEV_1_% 50–79 (*n* = 112)	8	20	72
3 FEV_1_% 30–49 (*n* = 53)	8	18	74
4 FEV_1_% < 30 (*n* = 13)	23	15	61

Table 3 shows the Cox univariable analysis that explores the independent association of each of the evaluated non-invasive CV risk factors with the risk of developing a CV event. The parameters that showed statistical significance were age, history of HTA, CRP levels, total cholesterol, LDL-cholesterol, CAC and the Framingham index. Interestingly, three well established noninvasive CV markers like Urine Albumin/Creatinine ratio, SCORE and EAT were not associated with the risk of developing a CV event. In Table 4, the multivariable analysis shows that CAC was the solely variable that remained in the model.

**Table 3:** Univariable analysis showing the independent association of each non-invasive CV risk marker with the risk of major CV event

Univariate analyses exploring factors that predict CV events		
Variable	HR (95% IC)	p
Age (year)	1.06 (1.03–1.08)	0.001
Pack-year (index)	1.01 (1.00–1.02)	0.003
BMI (index)	0.98 (0,94–1,03)	0,52
Gender (m/f)	0.59 (0.31–1.11)	0.10
Current Smoker (y/n)	0.53 (1.00–2.32)	0.04
FEV_1_%	0.99 (0.98–1.00)	0.07
Hypertension	2.53 (1.67–3.83)	0.001
DM	2.01 (1.22–2.32)	0.006
CRP (mg/dL)	1.24 (1.01–1.52)	0.03
Total Cholesterol (mg/dL)	0.99 (0.98–0.99)	0.007
HDL-C (mg/dL)	0.99 (0.98–1.00)	0.42
LDL-C (mg/dL)	0.98 (0.98–0.99)	0.001
Urine Albumin/Creatinine ratio	1.00 (0.99–1.00)	0.75
Framingham (for each %)	1.01 (1.00–1.03)	0.002
SCORE (for each %)	1.05 (0.98–1.11)	0.14
EAT (cm^3^)	1.00 (0.99–1.004)	0.27
Coronary Calcium Score (unit)	1.36 (1.24–1.48)	0.001

*BMI* Body Mass Index, *DM* Diabetes Mellitus, *HDL-C* High Density Protein, *LDL-C* Low Density Protein, *EAT* Epicardial Adipose Tissue

**Table 4:** The multivariable analysis indicated the Coronary Calcium score had a higher predictive power for risk of major CV event compared to the Framingham score

Multivariate analyses to exploring factors that predict CV events			
Variables	Coefficient	IC	p
Coronary Calcium Score (for each %)	1.32	1.19–1.46	0.001

## Discussion

The present study of patients with COPD attending our clinic shows that amongst the clinical, radiological and laboratory tools available to clinicians, the CAC score is the most powerful tool to evaluate the risk of suffering a major CV event in this high risk population.

It has been shown that additive models of individuals CV risk factors do not explain their interaction and synergy between variables and outcomes. Therefore, in the last decades, the concept of global risk assessment has been developed. One of the first tools to evaluate this global risk assessment was the one proposed by the Framingham study through its index [20]. Although this index has been extensively validated [22], it has several limitations. First, it only included patients from the United States not including other populations with different daily life activity and dietary habits. As an example, it has been shown that this index overestimates the CV risk in the European population [23]. To overcome this important limitation, European researchers have developed the SCORE study that validated the SCORE charts [18]. Interestingly, in our population of patients with COPD studied in Spain, the Framingham score showed statistical association, but only in the univariable analysis. Surprisingly the SCORE chart was not statistically significant even in the univariable analysis. Using the Framingham score, the estimated incidence of yearly CV events was 2.7% (27% estimate at 10 years), very similar to that observed after 5 years of follow up: 15% (3% per year). This confirms that COPD patients fall into the high-risk group (estimated 10 year CV risk > 20% for a mainly male cohort) for CV events. This is the first time the value of the Framingham score is validated in a COPD population.

Calcification of the coronary is an active process and can be seen in each of the stages of the development of an atherosclerotic plaque. The presence of calcium in the coronary artery is pathognomonic of coronary atherosclerosis, confirmed by histopathology and intravascular ultrasound studies [24–27]. Therefore, the CAC detected by chest CT is useful in estimating the burden of coronary atherosclerosis, and may be an effective tool for CV risk stratification in asymptomatic patients [21]. The absence of CAC in adults without evidence of CV disease excludes the presence of significant coronary atherosclerosis, which results in CV mortality rate of around 1% in 10 years [28, 29]. A recently published study by Williams et al. [30] confirmed that COPD patients have more CAC than controls and this is associated with increased dyspnea, reduced exercise capacity and increased general mortality. However, this study of the Evaluation of COPD Longitudinally to Identify Predictive Surrogate Endpoints (ECLIPSE) database only evaluated global mortality and not specifically CV deaths or CV events, and did not compare the predictive power of CAC with other well established noninvasive CV risk factors like cholesterol levels, CRP, microalbuminuria, Framingham score or SCORE charts. The current study shows that compared with other cardiovascular risk factors, the evaluation of CAC by a low dose chest CT is the best predictor of cardiovascular events in COPD patients (HR = 1.29; 1.08–1.56). This finding is important since to date, no information was available about the potential value of the CAC as a predictor of cardiovascular events in patients with COPD, and no data was available even in the general Spanish population. Results from the current study of COPD patients confirms that the presence of CAC is a good predictor of CV events, even when compared with the Framingham index [31–39].

The present study showed that elevated levels of CRP were associated with major CV events in the univariable analysis. Previously, Park et al. [40] studied 4905 adult males and showed that lower FEV_1_ was associated with higher levels of serum CRP, and a higher frequency of metabolic syndrome and coronary artery calcification on chest CT. However, in our study, the association between CRP level and CV events lost significance when compared with other noninvasive CV risk factors like CAC.

Interestingly, Romundstad et al. [41], exploring the Nord-Trøndelag Health Study (HUNT) database, found that MAB was associated with global mortality in COPD patients but not with CV mortality, as the present study did. These authors suggested that MAB could reflect the low grade systemic inflammation process, independent of the development of atherosclerosis.

Two unexpected findings from the present work were that SCORE and EAT were not associated with the development of CV event. As far as we can tell, this is the first time that these two well established noninvasive CV markers are specifically explored in a COPD population. They have been shown to predict CV outcomes in the general population but maybe for these specific markers we need a larger population and a longer follow up time if they are to be useful in patients with COPD.

The main strength of the present study is that it explored clinical, analytical and radiological non-invasive CV risk markers in a population of COPD patients prospectively followed for a long period of time. It also explored, for the first time, the potential utility of commonly used CV risk assessment tools like the Framingham index and the SCORE charts in this high risk population. The present study also had several limitations. Firstly, it was conducted in Caucasian patients with COPD followed in a tertiary center University Hospital and therefore the findings may not be applicable to the general population of patients with COPD. Secondly, a limited proportion of women were included in this study (although an equal opportunity to participate was offered) precluding the extrapolation of these findings to women with COPD. Thirdly, most patients included in the study had mild to moderate COPD, however, there is sufficient evidence showing that patients with mild to moderate COPD are the ones with higher risk of CV diseases [8, 42].

Our findings also convey a practical message. The use of CT scans of the thorax is increasing and this information may actually extends the use of such a tool, to help identify those subject within the COPD population were preventive measures such as smoking cessation, exercise promotion, detection and control of diabetes and dyslipidemia may help avoid subsequent CV events.

## Conclusions

In this cohort of patients with COPD from a pulmonary clinic of a tertiary University Hospital, CAC was the best predictor of CV events independently of other CV risk factors. If larger studies in different settings validate these findings, the incorporation of this tool to the practice of practitioners caring for patients with COPD could help detect high risk patients for future CV events.

## References

[1] Pauwels RA, Rabe KF (2004). Burden and clinical features of chronic obstructive pulmonary disease (COPD). Lancet.

[2] Sin DD, Anthonisen NR, Soriano JB, Agusti AG (2006). Mortality in COPD: role of comorbidities. Eur Respir J.

[3] Berger JS, Sanborn TA, Sherman W, Brown DL (2004). Effect of chronic obstructive pulmonary disease on survival of patient with coronary heart disease having percutaneous coronary intervention. Am J Cardiol.

[4] Sidney S, Sorel M, Quesenberry CP, DeLuise C, Lanes S, Eisner MD (2005). COPD and incident cardiovascular disease hospitalizations and mortality: Kaiser Permanente medical care program. Chest.

[5] Huiart L, Ernst P, Suissa S (2005). Cardiovascular morbidity and mortality in COPD. Chest.

[6] Hansell AL, Walk JA, Soriano JB (2003). What do chronic obstructive pulmonary disease patients die from? A multiple cause coding analysis. Eur Respir J.

[7] Mannino DM, Doherty DE, Buist AS (2006). Global Initiative on Obstructive Lung Disease (GOLD) classification of lung disease and mortality: findings from the Atherosclerosis Risk in Communities (ARIC) study. Respir Med.

[8] Calverley PM, Anderson JA, Celli B, Ferguson GT, Jenkins C, Jones PW, Yates JC, Vestbo J (2007). TORCH investigators. Salmeterol and fluticasone propionate and survival in chronic obstructive pulmonary disease. N Engl J Med.

[9] Anthonisen NR, Connett JE, Enright PL, Manfreda J (2002). Hospitalizations and mortality in the lung health study. Am J Respir Crit Care Med.

[10] Vogelmeier CF, Criner GJ, Martínez FJ, Anzueto A, Barnes PJ, Bourbeau J, Celli BR, Chen R, Decramer M, Fabbri LM, Frith P, Halpin DM, López Varela MV, Nishimura M, Roche N, Rodríguez-Roisin R, Sin DD, Singh D, Stockley R, Vestbo J, Wedzicha JA, Agustí A (2017). Global Strategy for the Diagnosis, Management, and Prevention of Chronic Obstructive Lung Disease 2017 Report: GOLD Executive Summary. Arch Bronconeumol.

[11] Chen W, Thomas J, Sadatsafavi M, FitzGerald JM (2015). Risk of cardiovascular comorbidity in patients with chronic obstructive pulmonary disease: a systematic review and meta-analysis. Lancet Respir Med.

[12] The Seventh Report of the Joint National Committee on prevention, detection, evaluation and treatment of high blood pressure. U. S. Department of Health and Human Services. National Institutes of Health. NHLBI. NIH Publication No. 03–5233. 2003.

[13] American Thoracic Society Statement (1991). Lung function testing; selection of reference values and interpretative strategies. Am Rev Resp Dis.

[14] Statement ATS (2002). Guidelines for the Six-Minute Walk Test. Am J Respir Crit Care Med.

[15] Mahler D, Weels C (1988). Evaluation of clinical methods for rating dyspnea. Chest.

[16] Celli BR, Cote C, Marin JM, Montes de Oca M, Mendez RA, Pinto Plata V, Cabral HJ (2004). The Body Mass Index, Airflow Obstruction, Dyspnea, Exercise Performance (BODE) index in chronic obstructive pulmonary disease. N Engl J Med.

[17] D'Agostino RB, Vasan RS, Pencina MJ, Wolf PA, Cobain M, Massaro JM, Kannel WB (2008). General cardiovascular risk profile for use in primary care: the Framingham Heart Study. Circulation.

[18] Conroy RM, Pyörälä K, Fitzgerald AP, Sans S, Menotti A, De Backer G, De Bacquer D, Ducimetière P, Jousilahti P, Keil U, Njolstad I, Oganov RG, Thomsen T, Tunstall-Pedoe H, Tverdal A, Wedel H, Whincup P, Wilhelmsen L, Graham IM (2003). Estimation of ten-year risk of fatal cardiovascular disease in Europe: the SCORE project. Eur Heart J.

[19] Sanchez-Salcedo P, Berto J, de-torres JP, Campo A, Alcaide AB, Bastarrika G, Pueyo JC, Villanueva A, Echeveste JI, Lozano MD, García-Velloso MJ, Seijo LM, García J, Torre W, Pajares MJ, Pío R, Montuenga LM, Zulueta JJ (2015). Lung cancer screening: fourteen year experience of the Pamplona early detection program (P-IELCAP). Arch Bronconeumol.

[20] Ding J, Hsu FC, Harris TB, Liu Y, Kritchevsky SB, Szklo M, Ouyang P, Espeland MA, Lohman KK, Criqui MH, Allison M, Bluemke DA, Carr JJ (2009). The association of pericardial fat with incident coronary heart disease: the Multi-Ethnic Study of Atherosclerosis (MESA). Am J Clin Nutr.

[21] Shemesh J, Henschke C, Shaham D, Yip R, Farooqi AO, Cham MD, McCauley DI, Chen M, Smith JP, Libby DM, Pasmantier MW, Yankelevitz DF (2010). Ordinal scoring of coronary artery calcifications on low-dose CT scans of the chest is predictive of death from cardiovascular disease. Radiology.

[22] Wilson PW, D’agostino R, Levy D, Belanger A, Sibershatz H, Kannel W (1998). Prediction of Coronary Heart Disease Using Risk Factor categories. Circulation.

[23] Motamed N, Rabiee B, Perumal D, Poustchi H, Miresmail SJ, Farahani B, Maadi M, Saeedian FS, Ajdarkosh H, Khonsari MR, Hemasi GR, Zamani F (2017). Comparison of cardiovascular risk assessment tools and their guidelines in evaluation of 10-year CVD risk and preventive recommendations: A population based study. Int J Cardiol.

[24] Rifkin RD, Parisi AF, Folland E (1979). Coronary calcification in the diagnosis of coronary artery disease. Am J Cardiol.

[25] Rumberger JA, Simons DB, Fitzpatrick LA, Sheedy PF, Schwartz RS (1995). Coronary artery calcium areas by electron beam computed tomography and coronary atherosclerotic plaque area: a histopathologic correlative study. Circulation.

[26] Schmermund A, Baumgart D, Adamzik M, Ge J, Grönemeyer D, Seibel R, Sehnert C, Görge G, Haude M, Erbel R (1998). Comparison of electron beam computed tomography with intracoronary ultrasound and coronary angiography for detection of coronary atherosclerosis. Am J Cardiol.

[27] Sangiorgi G, Rumberger JA, Severson A, Edwards WD, Gregoire J, Fitzpatrick LA, Schwartz RS (1998). Arterial calcification and not lumen stenosis is highly correlated with atherosclerotic plaque burden in humans: a histologic study of 723 coronary artery segments using non-decalcifying methodology. J Am Coll Cardiol.

[28] Budoff MJ, Achenbach S, Blumenthal RS, Carr JJ, Goldin JG, Greenland P, Guerci AD, Lima JA, Rader DJ, Rubin GD, Shaw LJ, Wiegers SE, American Heart Association Committee on Cardiovascular Imaging and Intervention, American Heart Association Council on Cardiovascular Radiology and Intervention, American Heart Association Committee on Cardiac Imaging, Council on Clinical Cardiology (2006). Assessment of coronary artery disease by cardiac computed tomography: a scientific statement from the American Heart Association Committee on Cardiovascular Imaging and Intervention, Council on Cardiovascular Radiology and Intervention, and Committee on Cardiac Imaging, Council on Clinical Cardiology. Circulation.

[29] Blaha M, Budoff MJ, Shaw LJ, Khosa F, Rumberger JA, Berman D, Callister T, Raggi P, Blumenthal RS, Nasir K (2009). Absence of coronary artery calcification and all-cause mortality. JACC Cardiovasc Imaging.

[30] Sarwar A, Shaw LJ, Shapiro MD, Blankstein R, Hoffmann U, Cury RC, Abbara S, Brady TJ, Budoff MJ, Blumenthal RS, Nasir K (2009). Diagnostic and prognostic value of absence of coronary artery calcification. JACC Cardiovasc Imaging.

[31] O’Malley PG, Taylor AJ, Jackson JL, Doherty TM, Detrano RC (2000). Prognostic value of coronary electron-beam computed tomography for coronary heart disease events in asymptomatic populations. Am J Cardiol.

[32] Williams MC, Murchison JT, Edwards LD, Agustí A, Bakke P, Calverley PM, Celli B, Coxson HO, Crim C, Lomas DA, Miller BE, Rennard S, Silverman EK, Tal-Singer R, Vestbo J, Wouters E, Yates JC, van Beek EJ, Newby DE, MacNee W; Evaluation of COPD Longitudinally to Identify Predictive Surrogate Endpoints (ECLIPSE) investigators. Coronary artery calcification is increased in patients with COPD and associated with increased morbidity and mortality. Evaluation of COPD Longitudinally to Identify Predictive Surrogate Endpoints (ECLIPSE) investigators. Thorax. 2014;69:718–723.

[33] Wong ND, Hsu JC, Detrano RC, Diamond G, Eisenberg H, Gardin JM (2000). Coronary artery calcium evaluation by electron beam computed tomography and its relationship to new cardiovascular events. Am J Cardiol.

[34] Raggi P, Cooil B, Callister TQ (2001). Use of electron beam tomography data to develop models for prediction of hard coronary events. Am Heart J.

[35] Kondos GT, Hoff JA, Sevrukov DML, Garside DB, Devries SS, Chomka EV, Liu K (2003). Electron beat tomography coronary artery calcium and cardiac events: a 37-month follow-up of 5635 initially asymptomatic low- to intermediate- risk adults. Circulation.

[36] Greenland P, LaBree L, Azen SP, Doherty TM, Detrano RC (2004). Coronary artery calcium score combined with Framingham score for risk prediction in asymptomatic individuals. JAMA.

[37] Taylor AJ, Bindeman J, Feuerstein I, Cao F, Brazaitis M, O'Malley PG (2005). Coronary calcium independently predicts incident premature coronary heart disease over measured cardiovascular risk factor: mean three-year outcomes in the Prospective Army Coronary Calcium (PACC) project. J Am Coll Cardiol.

[38] Budoff MJ, Shaw LJ, Liu ST, Weinstein SR, Mosler TP, Tseng PH, Flores FR, Callister TQ, Raggi P, Berman DS (2007). Long-term prognosis associated with coronary calcification. J Am Coll Cardiol.

[39] Detrano R, Guerci AD, Carr J, Bild DE, Burke G, Folsom AR, Liu K, Shea S, Szklo M, Bluemke DA, O'Leary DH, Tracy R, Watson K, Wong ND, Kronmal RA (2008). Coronary calcium as a predictor of coronary events in four racial or ethnic groups. N Engl Med.

[40] Raggi P, Gongora MC, Gopal A, Callister TQ, Budoff M, Shaw LJ (2008). Coronary artery calcium to predict all-cause mortality in elderly men and women. J Am Coll Cardiol.

[41] Arad Y, Goodman K, Roth M, Newstein D, Guerci AD (2005). Coronary calcification, coronary disease risk factors, C-Reactive protein, and atherosclerotic cardiovascular disease events. J Am Coll Cardiol.

[42] Blaha M, Budoff M, DeFilippis A, Blankstein R, Rivera JJ, Agatston A, O'Leary DH, Lima J, Blumenthal RS, Nasir K (2011). Associations between C-reactive protein, coronary artery calcium, and cardiovascular events: implications for the JUPITER population from MESA, a population-based cohort study. Lancet.

